# Novel Self-Cleaving Affinity Purification Method for Cellular Membrane-Associated Recombinant Paraoxonase-1 (rePON1) Enzyme

**DOI:** 10.1007/s10930-025-10271-y

**Published:** 2025-06-02

**Authors:** Milton S. Gonzalez-Serrano, Shuhan Chen, Alicia K. Friedman, Will Caines, Mason Pierce, Thomas J. Magliery, David W. Wood

**Affiliations:** 1 Department of Chemical and Biomolecular Engineering, The Ohio State University, Columbus, OH USA; 2 Department of Chemistry and Biochemistry, The Ohio State University, Columbus, OH USA

**Keywords:** Paraoxonase, Self-cleaving purification tag, Detergent additives, Tagless protein elution

## Abstract

Mammalian paraoxonase-1 (PON1) is a ~ 39.45 kDa calcium-dependent hydrolytic enzyme with potential therapeutic applications in chemical defense and cardiovascular disease. The N-terminus of PON1 is embedded in the cellular membrane, imparting to a hydrophobic character that leads to increased aggregation propensity and instability during purification. Although some advances have been made in bacterial expression hosts by using solubility-enhancing fusion tags and detergent solubilization strategies, these studies have shown that proteolytic tag removal is generally problematic. Thus, ineffective tag removal limits the bioanalytical characterization of the enzyme. Furthermore, the need for stabilizing detergents during purification limits the options for affinity-tag based methods. In this study, we demonstrate a novel affinity purification strategy by combining two solubility-enhancing fusion partners with the *i*CapTag™ self-removing affinity tag, where the entire purification process takes place in the presence of detergent. Optimization of purification conditions, including detergent and pH, resulted in the successful solubilization and stabilization of rePON1 at room temperature, allowing the tagless and native protein to be characterized. The results confirmed the expected catalytic efficiency and molecular weight of the enzyme. This method achieved over 95% host-cell protein impurities and more than 99.9% clearance of the host cell’s double-stranded DNA in a single-column affinity operation. This approach combines the power of affinity chromatography and facile tag removal, thereby offering a versatile and efficient alternative to produce other recombinant membrane-associated proteins, as well as additional target proteins that require challenging buffer conditions.

**Supplementary Information:**

The online version contains supplementary material available at 10.1007/s10930-025-10271-y.

## Introduction

Lipid bilayer membranes act as the main physical barriers to compartmentalize biochemical processes and maintain cellular homeostasis. Because these structures serve as cellular gateways, they also contain a plethora of signaling molecules that drive many vital metabolic pathways. In recent decades, membrane proteins have become essential targets for drug discovery and disease modeling [1–3]. Among these, membrane-associated receptors are targeted by about 60% of the approved drugs in the market [2, 3]. Applications that rely on these targets include the detection and treatment of chronic illnesses such as cancer, arthritis, diabetes, and additional autoimmune disorders [2, 3]. Thus, many biological drugs are designed to target the extracellular domains of membrane receptors, eliciting therapeutic responses through diverse modes of action that modulate downstream signaling pathways. Despite extensive efforts, membrane-associated proteins remain among the most challenging molecules to produce recombinantly, which can significantly slow the discovery and development of new drugs to target them.

PON1 is a membrane-associated mammalian hydrolytic enzyme that contains 354 amino acids (in humans) and is found on high-density lipoprotein (HDL) cholesterol particles in serum. At the center of PON1’s β-propeller structure, there is a central tunnel that houses two calcium ions that help maintain the structure. One of the Ca^2+^ ions that is exposed in the active site also plays a role in the enzyme’s catalytic activity [1]. The natural hydrolytic target of PON1 is unknown, but it is an efficient hydrolase of lactones and esters, and a weak hydrolase of organophosphates and other related compounds [4, 5].

Some pesticides, such as parathion, and nerve agents, such as sarin, are organophosphorus compounds that are often associated with accidental or intentional intoxication. Because of PON1’s weak organophosphatase (OPase) activity, it is a promising therapeutic lead against OP intoxication. In vivo studies have shown that mice injected with purified PON1 variants engineered for higher OPase activity exhibit increased protection against OP intoxication [6, 7]. Additionally, mice knocked out for PON1 develop atherosclerosis, suggesting that PON1 may also serve as an atheroprotective. Notably, Tward et al. found that PON1 could protect against atherosclerosis by preventing LDL oxidation [4]. High LDL oxidation has been correlated as a biomarker of atherosclerosis, and high PON1 activity has been found to lower the recurrence of symptoms in coronary heart disease [8–11].

Despite PON1’s therapeutic potential, it has proven difficult to express and purify [12]. Human PON1 is very challenging to produce even in mammalian cells [13], leading many studies to be carried out using a synthetic mammalian recombinant version (rePON1) produced by Tawfik and colleagues through the use of directed molecular evolution [14]. Despite this advance, the purification of rePON1 (also called G3C9) still poses significant challenges. Because its N-terminus is embedded in the cellular membrane (see supplementary Fig. S1) [15], it must be purified in the presence of detergent. Further, the affinity of rePON1 for Ca^2+^ is only in the micromolar range, so millimolar calcium salt is required to keep the protein stable and avoid aggregation. Previous work by Bajaj et al. [16, 17] also reports that hexahistidine (6xHis) recombinant affinity tags fused to either the N-terminus or C-terminus of rePON1 result in a significant decrease in enzymatic activity. Fusion of an N-terminal glutathione S-transferase (GST) domain improves soluble expression, but enterokinase cleavage of the tag is inefficient. Additional attempts in our own laboratories to express rePON1 with a C-terminal 6xHis tag reproduced the reduced activity observed by other groups, and these fusions also suffered from inefficient cleavage with tobacco etch virus (TEV) protease (SC and TJM, unpublished). Despite the advances in fusion-enhanced expression of rePON1 in bacterial systems, the required removal of the fused tags remains a major barrier to characterizing rePON1 enzymatic capabilities.

Inteins are protein segments that catalyze their excision from a host protein in a process called protein splicing. Split inteins are expressed into two separate protein polypeptides, and they can only exhibit splicing activity after association and folding into an active complex. This capability has been exploited for many applications in protein engineering and protein purification. To address the bottleneck of tag removal during the purification of various recombinant proteins, we have developed an affinity purification approach based on the commercially available *i*CapTag™ system. In this approach, the native intein splicing reaction has been modified to deliver isolated pH-sensitive cleaving at the C-terminus of the C-terminal segment of the assembled intein. The resulting *i*CapTag™ system consists of the N-terminal segment of an engineered split intein immobilized onto a conventional chromatography resin backbone, while the smaller (35 amino acid) C-terminal intein segment is expressed as a fusion tag on the N-terminus of the target protein (TP). Capture of the tagged target protein is carried out at an alkaline pH, enabling the isolation and purification of the TP under conditions that repress the cleaving reaction. Once purified, the assembled intein is induced to cleave and release the tagless target protein through a mild reduction in pH (typically pH 6 to 7), thus eliminating the need for proteolytic tag removal or harsh chemical treatments. Additionally, this method reduces the time and resources traditionally required for protein purification, making it an attractive choice for large-scale protein production, structural studies, or functional assays that might require high-throughput screening [18, 19].

Although the *i*CapTag™ intein system has been demonstrated for several globular recombinant proteins [18–20], there has been no demonstration of this approach for the purification of membrane-associated proteins. In the case of rePON1, specific considerations include difficulties in soluble expression, the loss of Ca^2+^ ions during purification that can lead to aggregation, and the required addition of detergent to stabilize the hydrophobic membrane-associated regions throughout the protocol. In addition, a loss of activity at slightly acidic pH (below 7), and enzyme inhibition were observed when using fused affinity or solubility tags at either terminus. In this work, we have addressed these issues through the development of optimized buffers and cleaving strategies for the rePON1 enzyme, which are compatible with the self-removing *i*CapTag™ system. The result is a highly purified, native and tagless rePON1 enzyme for biochemical characterization and further development.

## Experimental

### Buffer and Materials

All chemicals were purchased from Thermo Fisher or Sigma Aldrich unless otherwise stated. All the DNA cloning enzymes, including Q5 DNA polymerase, restriction enzymes, and Gibson Assembly master mix were purchased from New England Biolabs. All PCR primers were purchased from Sigma Aldrich. The trifluoroacetic acid (99% pure) used for titrating pH for mass spectrometry analysis was purchased from Alfa Aesar.

### DNA Plasmid Construction

**Fig. 1:**
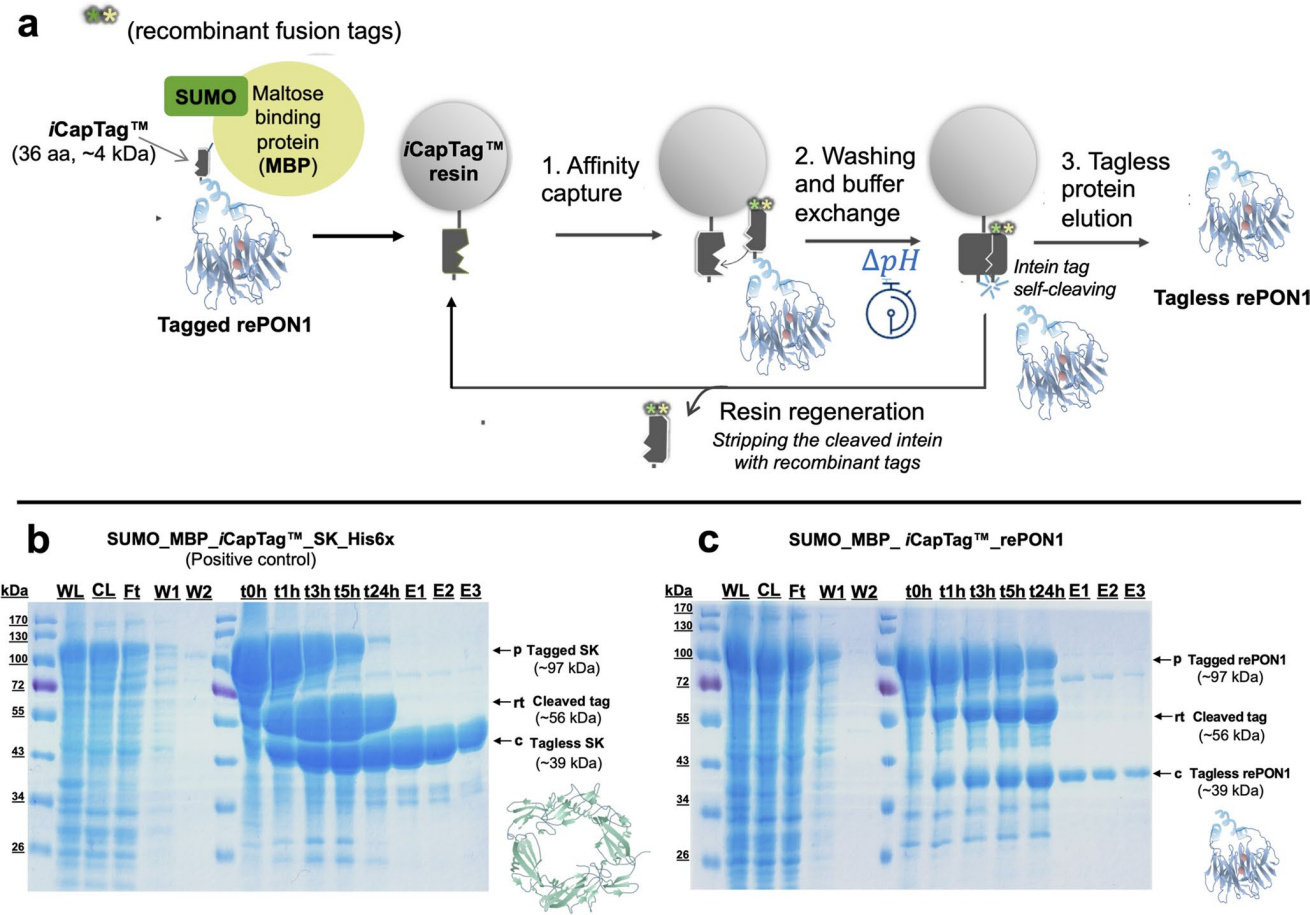
Npu split-intein-based purification trial with N-terminal SUMO-MBP recombinant fusion tags. **a** Self-cleaving affinity purification process overview; **b** SDS-PAGE analysis of different collected fractions of the purification for both streptokinase (SK) control, and **c** paraoxonase-1 (rePON1) target protein (PDB ID 3SRG shown in figure). Lanes: WL, whole lysate; CL, clarified lysate; W1, washing pH 8.5; W2, buffer exchange wash at pH 6.2; t0–24 h, boiled resin samples at given time points; and E1-3, elution fractions are 250 µL each

Small ubiquitin-like modifier (SUMO) and maltose binding protein (MBP) fusion tags were introduced at the N-terminus of *i*CapTag™ affinity tag to enhance the solubility of the rePON1 target protein (Fig. 1a). The resulting SUMO_MBP_*i*CapTag™ recombinant tag was then appended to the N-terminus of G3C9 paraoxonase-1 (rePON1) [19] by modifying the gene and inserting it into a pET32b vector with ampicillin resistance through Gibson assembly (New England Biolabs). The resulting expression plasmid is referred to as pET_SUMO-MBP_*i*CapTag™_rePON1. These recombinant tags were strategically introduced to enhance folding, solubility, and affinity capture of rePON1, respectively. As a positive control, the rePON1 gene was swapped with a streptokinase (SK) gene through Gibson Assembly (New England Biolabs) to evaluate the impact of fusion tags and detergent concentration on a well-behaved protein (pET_SUMO_MBP_*i*CapTag™_SK-His6x). To generate an enhanced cleaving mutant, the *i*CapTag™ intein fusion tag sequence was mutated to A33T through inverse PCR single-point mutation cloning. This mutation has been shown in previous work to significantly accelerate the cleaving reaction in some cases (data not shown), and it can be used with the commercially available *i*CapTag™ resin.

### Recombinant Expression of rePON1 Protein

Generated plasmids were transformed into *E. coli* Origami 2 (DE3) (Novagen) competent cells and grown for 16–18 h at 37 °C. Transformant colonies were cultured in 5 mL of 1 × Luria broth media (LB) with 200 µg/mL ampicillin final concentration. These cultures were grown at 37 °C in a shaking water bath at 220 rpm agitation for 16–18 h. The overnight cultures were then used to inoculate Thomson’s UltraYield™ flasks at 1% (v/v) in 100 mL of Terrific Broth media (TB, for 1 L of TB media, 12 g of Tryptone, 24 g of yeast extract, 4 mL of glycerol is added to deionized water, Q.S. to 900 mL and autoclaved; then 2.313 g KH_2_PO_4_, 12.54 g K_2_HPO_4_ is added to deionized water, Q.S. to 100 ml and autoclaved separately; once cooled, the solutions are mixed for the final medium) with 200 μg/mL ampicillin final concentration. Cultures were grown for 2–4 h until the OD_600_ reached at least 0.8–1.0 at 320 rpm agitation. The protein expression was then induced with isopropyl-beta-D-1-thiogalactopyranoside (IPTG) to a final concentration of 0.3 mM IPTG. The temperature was lowered to 30 °C and allowed to continue expression for 4–6 h.

### Cell Lysis and Sample Preparation

Cells were harvested by centrifugation at 8000 × *g* for 15 min at 4 °C. The culture supernatant was discarded, and the cells were then stored at − 20 °C or immediately resuspended in column buffer (25 mM Tris–HCl, 200 mM NaCl, 1 mM CaCl_2_, pH 8.5). Each cell pellet was resuspended with 1 mL of column buffer per 0.2 g of wet cell pellet and then was lysed with ten rounds of manual sonication with Virsonic 100 (Virstis) probe sonicator at 6–7 Watts for 30 s on ice, and 30 s resting. After sonication, a 30% v/v Tergitol (NP-10) detergent solution was added to a final concentration of 0.5% (v/v) of lysate and gently mixed with a pipette. The mixture was incubated for 1–1.5 h on ice. The lysates were then clarified via centrifugation at 21,000 × *g* for 15 min at 4 °C. The supernatants were then filtered using a 0.2 µm syringe filter. A 20 µL whole lysate sample and the supernatant were taken and mixed with 20 µL of 2x-SDS loading dye and heated to 98 °C for 5 min for SDS-PAGE analysis.

### *i*CapTag™ Intein Self-Cleaving Affinity Chromatography

While the samples were being incubated with the detergent on ice, 0.25 mL bead volume *i*CapTag™ resin (Protein Capture Science) was added to a 10 mL plastic gravity column (BioRad). Bottom caps were placed on each column, and the resin was regenerated by adding 10 CVs of regeneration buffer (6 M guanidine hydrochloride, 0.5 M NaCl). The top plug was sealed, and the columns were incubated for 1 h at room temperature while mixing on a spinning wheel. The columns were then drained and equilibrated with 5–10 CVs of pre-chilled column buffer with the selected ratio of v/v Tergitol (NP-10) detergent. The clarified lysates were then carefully applied to the settled resin and allowed to flow through, avoiding foam and bubble formation. In each case, the flowthrough fraction from column binding step was reapplied to the column two additional times. The resin was then washed with 10 CVs of the selected washing buffer (see Table 1), followed by 10 CVs of the corresponding elution buffer (see Table 1) to buffer exchange and shift the pH condition to trigger accelerated *i*CapTag™ tag self-cleavage on the column. Afterward, the bottom plugs were placed on each column, and one CV of elution buffer was added to the top of the settled bed to provide a 50% resin slurry. The column was then placed at room temperature for 24 h. At different time points, the 50% slurry resin was evenly resuspended by vortexing, and a 20 µL sample was taken and mixed with 20 µL of 2x-SDS loading dye and heated to 98 °C for 5 min. These samples were repeated as previously described at different time points to monitor the cleavage reaction progress via SDS-PAGE of the resin slurry. The column loading flow-through, wash 1, and wash 2 collected fractions were sampled as previously described for further SDS-PAGE analysis. After the 24-h resin sample, the columns were placed upright to allow the resin to settle by gravity, then the column was unplugged to collect the elution mobile phase into a labeled 1.5 mL Eppendorf microcentrifuge tube. Another CV of the selected elution buffer was then added to the column to recover residual tagless rePON1. This step was repeated two to three more times.

**Table 1:** Purification process buffer conditions screening

	Lysis step buffer	Equilibration step buffer	Washing step buffer	Cleavage and elution steps buffer
Condition 1	20 mM AMPD 20 mM PIPES 200 mM NaCl, 1 mM CaCl_2_ + 0.5% v/v Tergitol (NP-10) detergent; *added after sonication lysis*)) pH 8.5	20 mM AMPD 20 mM PIPES 200 mM NaCl, 1 mM CaCl_2_ 0.1% v/v Tergitol (NP-10) detergent pH 8.5	20 mM AMPD 20 mM PIPES 500 mM NaCl, 1 mM CaCl_2_ 0.1% v/v Tergitol (NP-10) detergent pH 8.5	20 mM AMPD 20 mM PIPES 200 mM NaCl, 1 mM CaCl_2_ 0.1% v/v Tergitol (NP-10) detergent pH 6.2
Condition 2	20 mM AMPD 20 mM PIPES 200 mM NaCl, 1 mM CaCl_2_ + 0.5% v/v Tergitol (NP-10) detergent; *added after sonication lysis*)) pH 8.5	20 mM AMPD 20 mM PIPES 200 mM NaCl, 1 mM CaCl_2_ 0.1% v/v Tergitol (NP-10) detergent pH 8.5	20 mM AMPD 20 mM PIPES 500 mM NaCl, 1 mM CaCl_2_ 0.1% v/v Tergitol (NP-10) detergent pH 8.5	20 mM AMPD 20 mM PIPES 200 mM NaCl, 1 mM CaCl_2_ 0.1% v/v Tergitol (NP-10) detergent pH 7.5
Condition 3	25 mM Tris HCl 200 mM NaCl, 1 mM CaCl_2_ + 0.5% v/v (added after sonication) Tergitol (NP-10) detergent; *added after sonication lysis*) pH 8.5	25 mM Tris–HCl 200 mM NaCl, 1 mM CaCl_2_ 0.3% v/v Tergitol (NP-10) detergent pH 8.5	25 mM Tris–HCl, 500 mM NaCl, 1 mM CaCl_2_ 0.3% v/v Tergitol (NP-10) detergent pH 8.5	25 mM Tris–HCl 150 mM NaCl 1 mM CaCl_2_ 0.3% v/v t Tergitol (NP-10) detergent pH 6.2
Condition 4	25 mM Tris HCl 200 mM NaCl, 1 mM CaCl_2_ + 0.5% v/v (added after sonication) Tergitol (NP-10) detergent; *added after sonication lysis*) pH 8.5	25 mM Tris–HCl 200 mM NaCl, 1 mM CaCl_2_ 0.3% v/v Tergitol (NP-10) detergent pH 8.5	25 mM Tris–HCl, 500 mM NaCl, 1 mM CaCl_2_ 0.3% v/v Tergitol (NP-10) detergent pH 8.5	25 mM Tris–HCl 150 mM NaCl 1 mM CaCl_2_ 0.3% v/v t Tergitol (NP-10) detergent pH 7.5
Condition 5	25 mM Tris HCl 200 mM NaCl 1 mM CaCl_2_ + 0.5% v/v Tergitol (NP-10) detergent; *added after sonication lysis*)) pH 8.5	25 mM Tris–HCl 200 mM NaCl 1 mM CaCl_2_ 0.3% v/v Tergitol (NP-10) detergent pH 8.5	25 mM Tris–HCl, 500 mM NaCl 1 mM CaCl_2_ 0.3% v/v Tergitol (NP-10) detergent pH 8.5	25 mM Tris–HCl 150 mM NaCl 1 mM CaCl_2_ 0.3% v/v Tergitol (NP-10) detergent pH 8.0

### Sample Analysis

#### SDS-PAGE and Densitometry Analysis

The whole lysate, clarified lysate, column loading flow-through, washing 1, washing 2, time point resin samples, and elution fractions from the *i*CapTag™ cleaving reaction were analyzed by SDS-PAGE. Gels were loaded with equal volumes of protein samples. SDS-PAGE was performed on 8% polyacrylamide resolving gels to view the mass shift of cleaved rePON1. These were electrophoresed in 1 L of running buffer (1 × Tris–glycine) at a constant 180 V until the bromophenol blue dye ran off the gel. The gels were then removed from plates and stained with Coomassie brilliant blue G-250 dye. The SDS-PAGE gel images were scanned, and densitometry analysis was performed using ImageJ software to estimate the purity. The protein concentration was estimated with a SpectraMax QuickDrop UV–Vis spectrophotometer (Molecular Devices) with the specific molar extinction coefficient at 280 nm for tagless rePON1 (44,350 M^−1^ cm^−1^) computed by the SnapGene software package (https://www.snapgene.com).

#### Double-Stranded DNA Quantification Assay

Samples from the clarified lysates and the final purification elution were submitted to the OSU Genomic facilities for double-stranded DNA quantification analysis with the Qubit™ double-stranded DNA HS quantification assay kit (Invitrogen). Results were reported in concentration (ng/µL). Host cell DNA clearance was then calculated as a difference percentage with Eq. (1).

1 $$ \begin{aligned} & {\text{Host }}\;{\text{Cell }}\;{\text{DNA}}\;{\text{ Clearance}} \\ & \quad = \left( {\left[ {{\text{Final }}\;{\text{Elution}}\;{\text{dsDNA}}} \right]/[{\text{Cell }}\;{\text{Lysate }}\;{\text{dsDNA}}]} \right)*100\% \\ \end{aligned} $$

### MALDI-TOF Mass Spectrometry analysis

Samples were prepared for MALDI-TOF analysis using the dried droplet method with sinapic acid as the matrix. Briefly, a 1:1 mixture of protein sample and matrix solution (sinapic acid at a concentration of 10 mg/mL dissolved in 50% acetonitrile, 50% water, and 0.1% TFA) was prepared. About 0.5 µL of this mixture was spotted onto a polished steel target plate and allowed to crystallize via evaporation at room temperature. Proteins were characterized by MALDI-TOF mass spectrometry using a Bruker Microflex LRF system (Bruker Daltonics, Inc., Billerica, MA, USA) in linear detection mode. All spectra were acquired in positive-ion mode from > 800 summed laser shots randomly distributed throughout the sample spot. BSA (bovine serum albumin, Sigma Aldrich) was used as an external mass calibrant from adjacent spot locations to the sample spots. Spectra were processed and visualized via default parameters using the FlexAnalysis software (version 1.4, Bruker Daltonics, Inc.).

### Enzyme Activity Assay

Phenyl acetate (Sigma-Aldrich) was used as the substrate to measure the enzymatic hydrolysis activity [13]. It was first dissolved to stock concentrations of 1–100 mM using methanol. Then, 186 μL of 50 mM NaCl, 10 mM HEPES (pH 7.0) buffer, 4 µL substrate stocks, and 10 µL enzyme (adjusted to 1.4 nM rePON1 final concentration) were added to each well of Greiner UV-Star 96 well plates so that all reactions could be carried out at the same concentration of methanol (2%). No additional calcium ion was added beyond what was carried in the elution buffers shown in Table 1. Reaction rates were measured with a SpectraMax M5 (Molecular Devices) at 270 nm at room temperature, using an extinction coefficient of 1310 M^−1^ cm^−1^ for phenol. One reading was taken every 10 s. The initial linear region (with < 10% substrate consumed) was used to obtain the initial reaction rates, and both the Michaelis–Menten Equation (Eq. 2, Kaleidagraph) and a linear model (Microsoft Excel) with Eq. (3) were fit to the initial substrate concentration versus initial rate data.

2 $$ {\text{Michaelis}} - {\text{Menten equation}};\,v_{0} = v_{max} \left[ S \right]_{0} /\left( {K_{m} + \left[ S \right]_{0} } \right) $$

3 $$ v_{0} \approx \left( {v_{max} /K_{m} } \right)\left[ S \right]_{0} \,\,\,\,\,{\text{when}}\left[ S \right]_{0} < < K_{m} $$

## Results

### Protein Expression and *i*CapTag^TM^ Purification Test

To enhance the solubility of rePON1, SUMO and MBP solubility-enhancing tags were fused to the N-terminus of the *i*CapTag™ affinity purification tag. To assess the impact of these modifications on protein expression and purification, streptokinase (SK), a well-characterized and highly soluble target protein, was used as a model control protein. In this case, the combination of SUMO and MBP provide a large enough tag to observe a clear mass shift during the cleavage reaction. The *i*CapTag™ self-cleaving affinity purification process was carried as described in Fig. 1a. As shown in Fig. 1b and c, the SUMO-MBP tagged proteins show appreciable solubility and remain in the clarified lysate fraction for both rePON1 and SK. Resin samples taken at time zero (Fig. 1b and c, lanes t0h) indicate significant binding of each protein to the *i*CapTag™ resin, indicating that the SUMO-MBP tag does not negatively impact the affinity capture step. Additional resin samples at later timepoints confirm that the *i*CapTag™ self-cleavage reaction is not abolished by the presence of these tags and proceeds at typical rates observed in previous work. Although reports in the literature have shown inhibition of intein activity in the presence of divalent metals such as Zn^+2^, Cu^+2^, and Co^+2^ [21, 22], preliminary experiments in this work have shown that this *i*CapTag™ intein can tolerate up to 5 mM CaCl_2_ without significant loss in activity (see supplementary Fig. S2). The SK-His6x control protein was able to reach ~ 97% cleavage in 24 h with 100% recovery and over 91% purity upon elution (Fig. 1b). On the other hand, rePON1 showed around 32% cleavage with incomplete elution of the cleaved product from the resin at 24 h (Fig. 1c), which usually indicates some aggregation of the cleaved product on the affinity resin. This indicates that the elution buffer conditions should be optimized for rePON1 to improve solubility.

### Elution Buffer Conditions Screening

**Fig. 2:**
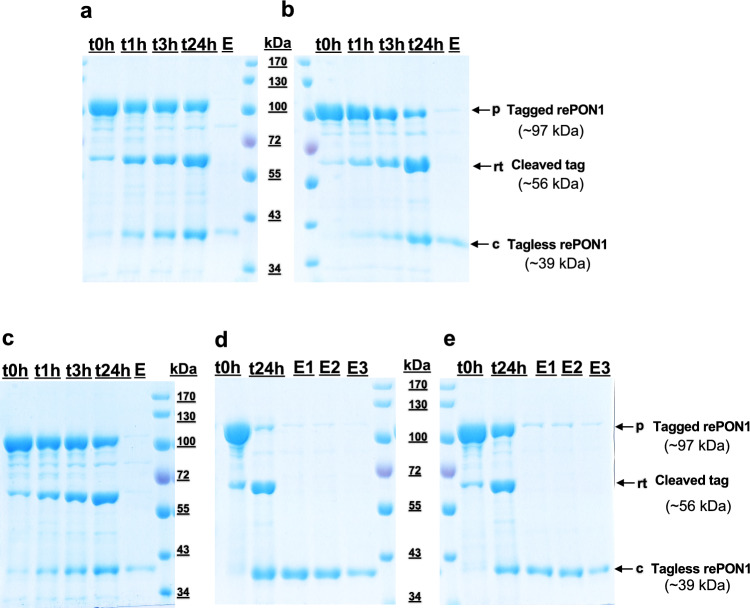
Elution buffer condition screening with different pH and Tergitol NP-10 detergent concentrations. Time course tag-cleavage reaction on the *i*CapTag™ resin, sampled at 0, 1, 3, and 24 h, followed by elution under the indicated conditions (corresponding to conditions 1 to 5 shown in Table 1): **a** 0.1% (v/v) Tergitol at pH 6.2; **b** 0.1% (v/v) Tergitol at pH 7.5; **c**, 0.3% v/v Tergitol at pH 6.2; **d** 0.3% (v/v) Tergitol at pH 7.5; and **e** 0.3% (v/v) Tergitol at pH 8.0. The t0–24 h, boiled resin samples at given time points; and E1-3, elution fractions are 250 µL each

Based on previous analysis showing an impact of pH and detergent in protein stability, the elution buffer pH and different detergent concentrations were evaluated. Preliminary work has shown that a single point mutation on the *i*CapTag™ intein affinity tag (A33T, data not shown) can enhance cleavage activity at higher pH levels, allowing it to function effectively over a broad range of pH values (MSGS and DWW, manuscript in preparation). This modification was introduced to accommodate tag cleavage at higher pH values of 7.5 and 8.0, as described in Table 1. In the initial test run, pH 6.2 and 7.5 values were evaluated with 0.1% (v/v) Tergitol detergent. The pH 6.1 with 0.1% v/v Tergitol was able to reach about 31% cleavage, while the pH 7.5 with 0.1% v/v Tergitol only reached ~ 24%. Although rePON1 was able to cleave under both conditions 1 and 2 (pH 6.2 and 7.5 with 0.1% Tergitol each), poor recovery was observed (Fig. 2a and b) with weaker rePON1 elution bands via SDS-PAGE analysis. In the next trial, the pH in the elution buffer was then kept at pH 6.2, while the Tergitol concentration was adjusted to 0.3% (v/v) (condition 3, Table 1) to evaluate if the more detergent can help maintain the tagless rePON1 solubility in the aqueous phase. Despite the increased detergent concentration, results were similar to the previous experiment as it reached nearly 36% cleavage, but only a small fraction of the tagless rePON1 band was visible in the elution (Fig. 2c).

While the 0.5% v/v Tergitol detergent addition in the lysis step helped to enhance the solubilization of rePON1, the pH condition in this step was at ~ 8.5. Therefore, additional experiments were performed to test a combination of higher pH conditions with more Tergitol detergent. In this case, rePON1 in pH 7.5 with 0.3% (v/v) Tergitol (condition 4, Table 1) was able to cleave ~ 89.2% after a 24-h incubation at room temperature. This condition showed appreciable recovery with comparable protein band intensities between the 24-h resin sample and the elution mobile phase sample on the SDS-PAGE analysis (Fig. 2d). Furthermore, the purity of the eluted product in this method was estimated to be around ~ 97.4%(Fig. 2d). Interestingly, the pH 8.0 buffer (condition 5, Table 1) shows similar protein band intensity patterns between the resin sample at 24 h and the eluted tagless rePON1, although the cleavage achieved was around 40% (Fig. 2e). These results suggest that the cleaved protein requires a higher pH to remain soluble in the elution buffer. The theoretical isoelectric point of rePON1 determined from SnapGene software was around pH ~ 6.6, which could explain the observed poor recovery at pH 6.2. Based on these screening experiments, condition 4 was selected for subsequent purifications.

### Purification Analysis

**Fig. 3:**
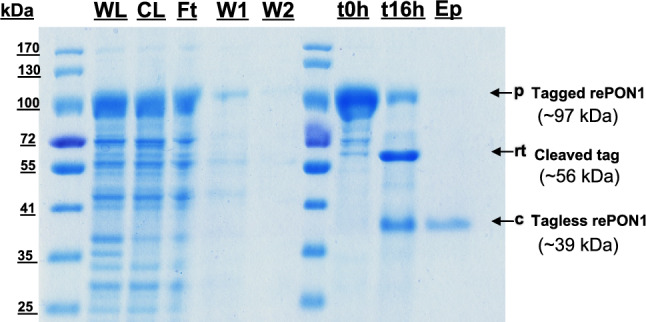
SDS-PAGE of the rePON1 purification with optimized elution buffer including 0.3% v/v Tergitol detergent at pH 7.5, cleaved for 16 h. Lanes: WL, whole cell lysate; CL, clarified lysate; Ft, column binding flowthrough; W1, washing buffer; W2, buffer exchange wash at pH 7.5; t0–t16h, time point resin samples; Ep, pooled elution fractions 1–6 (250 µL each)

The samples were purified in duplicate using condition 3. The *i*CapTag™ is compatible with a variety of detergents (see supplementary Fig. S2c–e), allowing flexibility in detergent choice and concentration without compromising cleaving activity. In the selected elution buffer condition, the protein can reach ~ 77.6% cleavage in 16 h (shorter than the 24 h shown above), with very pure tagless rePON1 shown by the protein band in the elution mobile phase (Fig. 3). The shorter cleaving time was selected to minimize the period of incubation at room temperature during the cleaving reaction. Under these conditions, the estimated average rePON1 yield from the pooled elution fractions was about 218.1 ± 8.3 µg of rePON1 from 30 mL of shake flask volume using 200 µL of *i*CapTag™ resin bed volume (see Table 2). Additionally, the estimated purity was ~ 97.4%, thus offering tagless rePON1 with high purity.

**Table 2:** *i*CapTag™ purification analysis summary

Average intein cleavage (%)	Pool elution conc. (µg/mL)	Average rePON1 yield (µg)	Average rePON1 yield moles (nmol)	rePON1 estimated binding capacity to *i*CapTag™ resin	Average purity (%) based on the SDS-PAGE densitometry analysis	Average host cell dsDNA conc. clarified lysate (ng/µL)	Average dsDNA conc elution fraction (ng/µL)	dsDNA clearance (%)
~ 77.6	145.4 ± 5.5	218.1 ± 8.3	5.5 ± 0.14	~ 0.874 mg of rePON1 per mL of resin bed vol	~ 97.4	> 120	< 0.1	> 99.9

n = 2

Host cell DNA clearance is considered a critical quality attribute in the manufacture of recombinant therapeutic proteins and was therefore evaluated in this study for rePON1. The Qubit double-stranded (ds) DNA quantification assay reported concentrations > 120 ng/µL dsDNA for the clarified lysate fraction and < 0.1 ng/µL dsDNA for the purification elution. Based on these results, the *i*CapTag™ intein-based purification method has the capability of clearing more than 99.9% of the host-cell DNA (see Table 2), not only providing a tagless rePON1 but also is highly pure.

The eluted protein samples were submitted for MALDI-TOF analysis in the OSU Department of Chemistry and Biochemistry mass spectrometry facility. This analysis was performed in the same elution buffer as Condition 4 (Table 1) but without sodium chloride. Because of the hydrophobic nature of this enzyme, it becomes unstable and precipitates in more common mass spectrometry-compatible buffers. The analysis was carried out by spotting the sample matrix directly onto the plate. The spectra show a base peak of 39,256 Da (Figure S3), which is consistent with the computed molecular weight of tagless rePON1 (39,451 Da using SnapGene) within the expected error of MALDI TOF used without internal calibration. The purification analysis experiments confirm that the elution contains very pure tagless rePON1.

### Enzyme Activity Assay

**Fig. 4:**
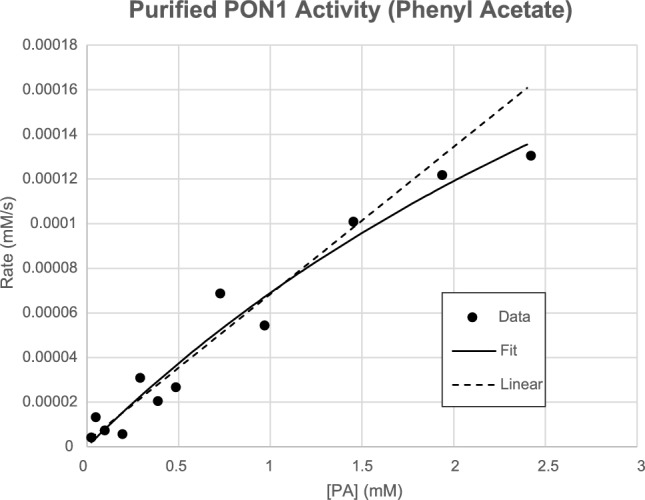
Enzyme activity. A plot of the rate of hydrolysis of phenyl acetate by purified rePON1 at varying concentrations of substrate. Each rate is the average of three trials. Michaelis–Menten (solid line) and linear (dashed line) models are shown

The activity of the purified rePON1 was assayed by following the hydrolysis of phenyl acetate (PA), calculated from a change in absorbance at 270 nm. Although paraoxonase-1 was named for its ability to hydrolyze paraoxon, its paraoxonase activity is low; its best substrates are lactones and aryl esters. We followed the initial rate of hydrolysis at concentrations of PA from 0.024 to 2.40 mM, all at a constant 2% methanol. Rates were computed from the average of three trials (Data provided in the supplemental Fig. S4). Fitting the Michaelis–Menten equation to the data gives a *k*_*cat*_ of 300 ± 100 s^−1^ and a *K*_*M*_ of 5 ± 3 mM (Fig. 4). The fitted value for *K*_*M*_ lies outside the tested range of values, which leads to high error for this estimate. The *k*_*cat*_ is within error of the previously reported value of 350 s^−1^ [23]. However, because the *K*_*M*_ value for PA is high, the enzyme was not saturated in this experiment, and so the error in *k*_*cat*_/*K*_*M*_ is high (~ 100%). The *k*_*cat*_/*K*_*M*_ can be estimated better from the slope of a linear fit up to 1.45 mM PA, which gives a value of 47,000 ± 2000 M^−1^ s^−1^ (Fig. 4). These data suggest that folded, active rePON1 is produced from our optimized purification.

## Discussion

The rePON1 enzyme, like many other membrane-associated proteins, presents a challenge for downstream purification and processing. Every protein requires an expression optimization and solubilization study to ensure effective extraction of the protein into the aqueous phase. Combining SUMO-MBP fusion tags with Tergitol detergent was critical for efficiently expressing and extracting rePON1 from the lysates. As shown in this work, rePON1 needed a higher concentration of 0.3% v/v/ Tergitol detergent in the purification buffers than what was initially attempted (0.1% v/v) with conditions 1 and 2 (Table 1). Even though detergents are critical for solubilizing hydrophobic proteins, they don’t necessarily guarantee protein sample stability, as proteins in detergent micelles are often unstable [24]. Increasing detergent in the purification buffers helped to maintain rePON1 solubility by promoting more effective formation of micelle structures around the hydrophobic region on the protein [24, 25]. This enhances the coverage of rePON1’s exposed hydrophobic N-terminus (H1) and membrane-interacting (H2) alpha helices (See supplemental Fig. S1). In this case, the H1 and H2 functions as the rePON1 membrane anchor [15].

Although buffer pH is an important factor for any kind of protein, it was evident that rePON1 required a higher pH during purification to prevent precipitation after *i*CapTag™ cleavage. In this case, the initial *i*CapTag™ cleavage reaction was carried out at a pH of 6.2, which is not too distant from rePON1’s isoelectric point (pH ~ 6.6) and can promote protein aggregation on-column. To address this issue, the buffer pH was increased to 7.5, which decreases the cleaving kinetic rates (data not shown). A 3 × higher concentration of Tergitol combined with pH ≥ 7.5 with 0.3% v/v Tergitol exhibited the best results for solubilization. The pH 8.0 with 0.3% v/v Tergitol (Fig. 2e) demonstrated comparable protein band intensities of the elution sample to resin samples at 24 h to those in pH 7.5 with 0.3% v/v Tergitol condition (Fig. 2d). In this case, the A33T mutation on the *i*CapTag™ affinity tag enhanced intein cleavage and allowed the reaction to reach about 89% cleavage under pH 7.5 and ~ 40% for pH 8.0.

A study by P Bajaj et al. [26] reported a recombinant rePON1 solubilization with 2% v/v Triton X-100 from bacterial inclusion bodies and its purification with traditional immobilized metal affinity chromatography (IMAC). However, the yields in their study were substantially low, and they therefore focused on recovering the inclusion bodies and using a refolding strategy [26]. In contrast, our work shows that combining solubility-enhancing tags and 0.3% v/v Tergitol detergent can suffice for effective solubilization without the need to recover from inclusion bodies [14, 26]. In addition, the *i*CapTag™ resin offers a binding capacity of at least 0.874 mg of soluble tagless rePON1 per mL of resin bed volume (the resin was likely not saturated in this study). This confirms that the purification process can combine solubility-enhancing tags with reduced detergent concentrations to achieve successful solubilization and purification of rePON1, eliminating the need for additional refolding steps that further complicate processing.

In addition, the *i*CapTag™ purification method shows about 97.4% purity and more than 99.9% host-cell DNA clearance, overall showing high purity. The identity of the protein was confirmed by MALDI-TOF mass spectrometry and was verified with an enzyme activity assay. Results showed tagless rePON1 to have phenyl acetate hydrolysis activity with an estimated catalytic efficiency of 47,000 ± 2000 M^−1^ s^−1^ (Fig. 4). This value is within ~ 3x-fold less than the previously reported value for G3C9 rePON1 (~ 1.5 M^−1^ s^−1^), which was purified with the IMAC method via a 6xHis tag on the C-terminus [23]. Overall, this enzyme assay shows that the generated tagless rePON1 is active. This optimization experiment not only demonstrates the challenges of working with recombinant membrane-associated proteins, but that it is feasible for the self-cleaving *i*CapTag™ purification method in different buffer conditions.

As demonstrated by the experimentation detailed above, tagless recombinant rePON1 can be purified using Npu split intein-based self-cleaving affinity purification technology (*i*CapTag™). Buffers and tag optimization yielded ~ 97% pure product with over 99.9% host cell dsDNA clearance. This single-column purification step not only offers a highly effective affinity capture, but can also accommodate a variety of buffer compositions while maintaining the capabilities for on-column cleavage of recombinant fusion tags. Among the major advantages of this method is that it eliminates the need for a fusion tag removal step and provides an opportunity to elute proteins directly into assay and formulation buffers. This successful purification of rePON1 marks the first example of a membrane-associated protein purified using the *i*CapTag™ system in the literature, demonstrating that this novel technology is robust and can likely be expanded to other recombinant membrane protein-based therapeutics and targets.

## Supplementary Information

Below is the link to the electronic supplementary material.

Supplementary file1 (DOCX 3836 KB)

## Data Availability

Data is available from the authors by request.
