# Supplementary material for: Novel Self-Cleaving Affinity Purification Method for Cellular Membrane-Associated Recombinant Paraoxonase-1 (rePON1) Enzyme
Source: Protein J. 2025 Jun 2;44(4):341–50. doi: 10.1007/s10930-025-10271-y (PMC12313795; doi:10.1007/s10930-025-10271-y)
Supplement: Supplementary file 1 — Supplementary file1 (DOCX 3836 KB) [file 10930_2025_10271_MOESM1_ESM.docx]

**Appendix. Supplementary information**

Npu self-cleaving affinity chromatography

While the samples were incubated with the detergent on ice, 0.25 mL bead volume *i*CapTag^(TM)^ resin (Protein Capture Science) was packaged in a 10 mL plastic gravity column (BioRad). The bottom plugs were placed on each column, and then the resin was regenerated by adding 10 CVs of regeneration buffer (6 M guanidine hydrochloride, 0.5 M NaCl). The top plug was sealed, and the columns were incubated for 1 hr. at room temperature while mixing on a spinning wheel. The columns were then drained and then equilibrated with 5-10 CVs of pre-chilled column buffer with different detergents (0%, 5% v/v Tween20, and 5% v/v TritonX100). The lysates were then carefully applied, avoiding foam and bubble formation. The resin was washed with 10 CVs of washing buffer (25 mM Tris, 500 mM NaCl, with 0% and 5% v/v for the respective detergents, all at pH 8.5), followed by 10 CVs wash with elution buffer (25 mM Tris, 150 mM NaCl, a with 0% and 5% v/v of the respective detergents at pH 6.2) to buffer exchange and shift the pH to trigger accelerated Npu_C_ tag self-cleavage on column. Afterward, the gravity bottom plugs were placed on each column, and one CV of elution buffer was added and placed at room temperature for 24 hrs. The 50% slurry resin was evenly resuspended with the vortex, and a 20 µL sample was taken and mixed with 20 µL of 2x-SDS loading dye and boiled at 98 °C for 5 minutes to quench the cleavage reaction. These samples were repeated as previously described at different time points to monitor the cleavage reaction progress for the first five hours of the reaction. The capture flow-through, washing 1, and washing 2 collected fractions were sampled as previously described for further SDS-PAGE analysis.

SDS-PAGE and densitometry analysis

The whole lysate, clarified lysate, capture flow-through, washing 1, washing 2, time point resin samples, and elution fractions from the Npu affinity chromatography were analyzed by SDS-PAGE. Gels were loaded with even volumes of SDS samples. One-dimensional SDS-PAGE was performed on 8% acrylamide resolving gels to appreciate the mass shift of cleaved rePON1. These were run in 1 L of running buffer (1x Tris-glycine) at a constant 180 V until the blue dye ran off the gel. The gels were then removed from plates and stained in Coomassie brilliant blue G-250 dye. The SDS-PAGE gel images were captured with a scanner. The densitometry analysis was performed using ImageJ software to estimate the purity

**Figure S1.** The rePON1 composition. (a) rePON1 protein sequence with alpha helices motifs H1 and H2 hydrophobic regions highlighted in light blue [15]. (b) The rePON1 predicted structure in the Alphafold database with alpha helices H1 and H2 hydrophobic regions highlighted in light blue (structure model AF-A0A667GCF1-F1-v4).

**Figure S2**. Npu self-cleaving affinity purification test on Npu_C_ tagged eGFP to qualitatively verify intein binding and cleaving activity in the presence of different detergents. Purification experiment workflow (a), NpuC tagged eGFP capture and cleavage (b), SDS-PAGE analysis for purification process with different time point resin samples with no detergent additives (c), 5% v/v tween20 detergent (d), 5% v/v triton X100 (e), and 5% v/v Tergitol with 5 mM CaCl_2_ (F).

**Figure S3.** MALDI-TOF spectra for tagless rePON1 pooled elution fractions.


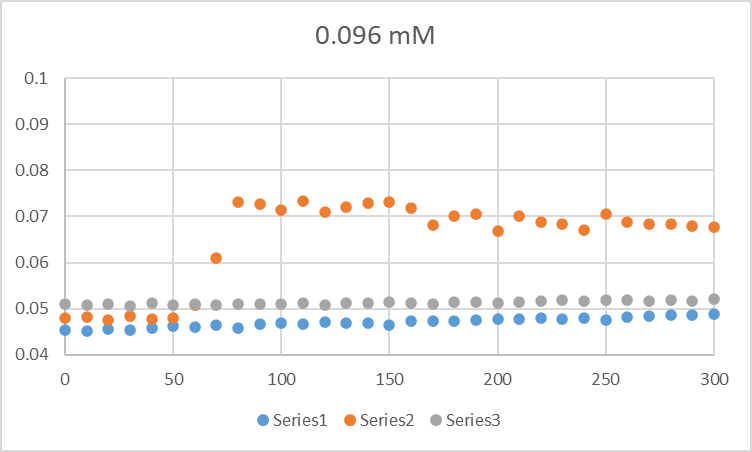


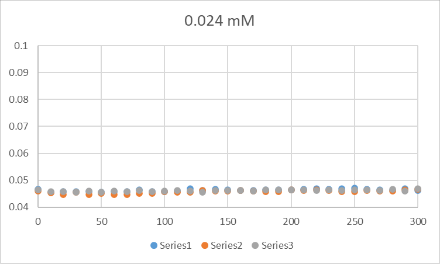

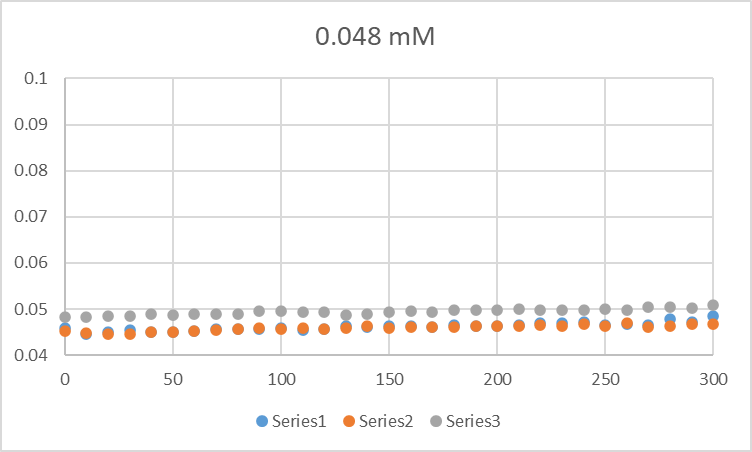


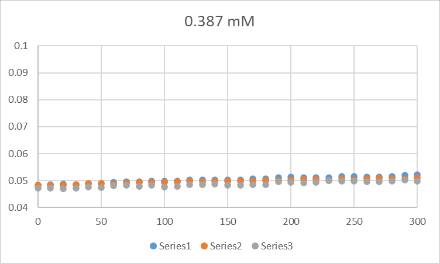

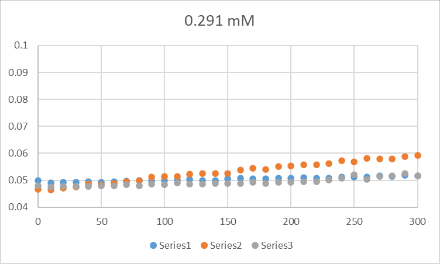

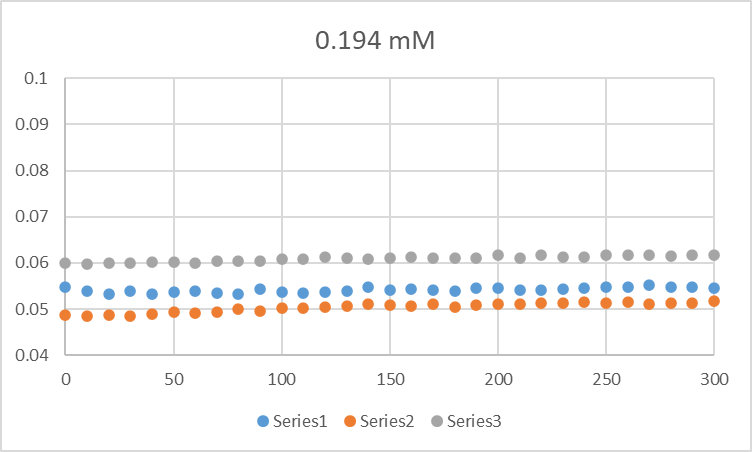


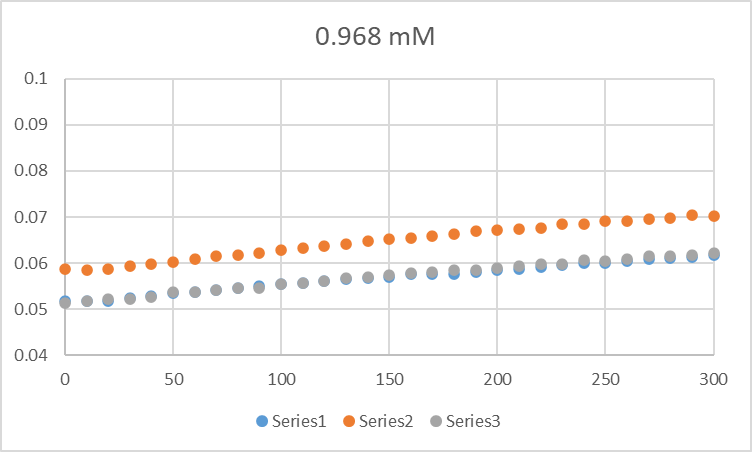

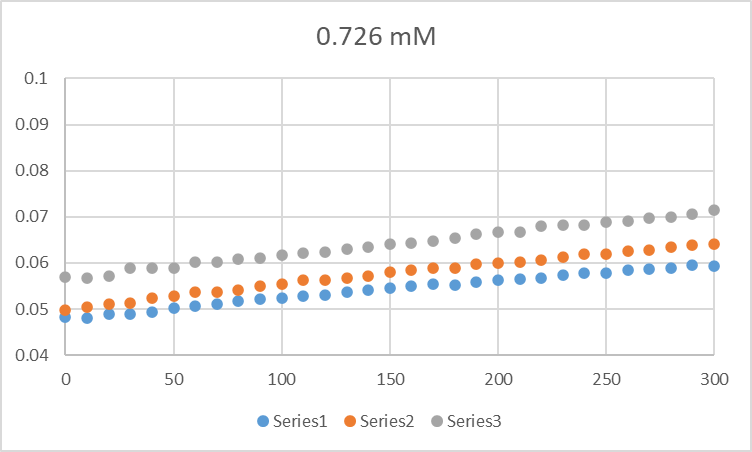

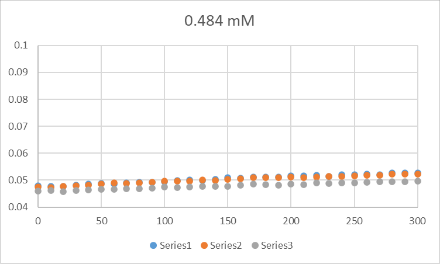


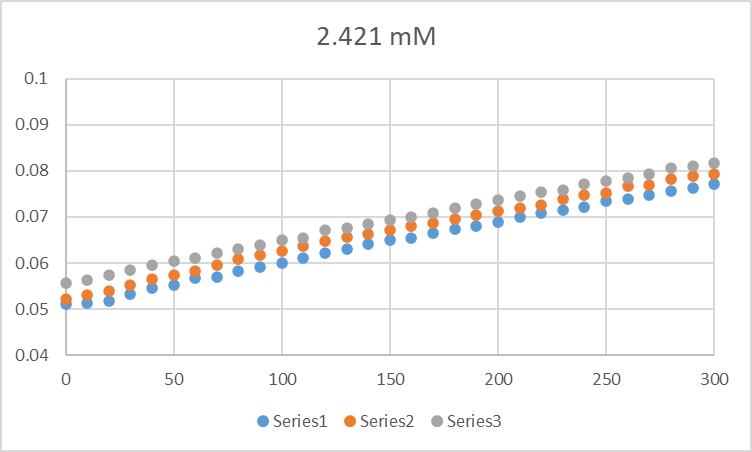

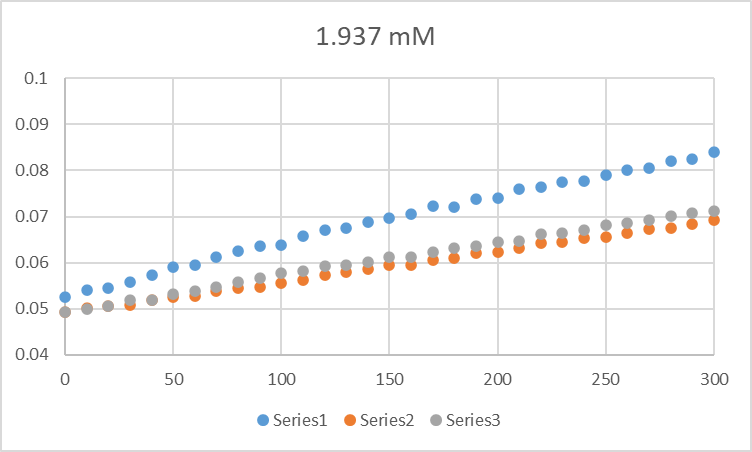

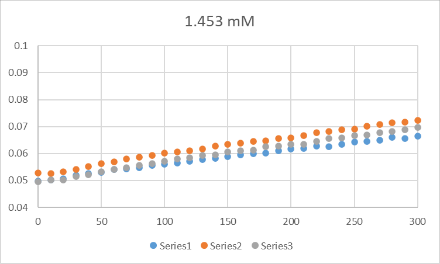


**Figure S4.** The raw data are shown with times in seconds on the *x*-axis and absorbance at 270 nm on the *y*-axis. The concentration of phenyl acetate for each time point is shown. Separately, a no-enzyme blank for each condition was also run and subtracted from these values for fitting (not shown).
